# Supplementary material for: Mirtazapine Reduces Adipocyte Hypertrophy and Increases Glucose Transporter Expression in Obese Mice
Source: Animals (Basel). 2020 Aug 14;10(8):1423. doi: 10.3390/ani10081423 (PMC7459487; doi:10.3390/ani10081423)
Supplement: Supplementary file 1 [file animals-10-01423-s001.pdf]

animals

MDPI

Article

Mirtazapine Reduces Adipocyte Hypertrophy and Increases Glucose Transporter Expression in Obese Mice

Ching-Feng Wu [1,†], Po-Hsun Hou [2,3,†], Frank Chiahung Mao [4], Yao-Chi Su [5], Ching-Yang Wu [1], Wei-Cheng Yang [6], Chen-Si Lin [6], Hsiao-Pei Tsai [5,7,8], Huei-Jyuan Liao [5] and Geng-Ruei Chang [5,7,*]

1 Division of Thoracic and Cardiovascular Surgery, Department of Surgery, Chang Gung Memorial Hospital, Chang Gung University, Linkou, 5 Fuxing Street, Guishan District, Taoyuan 33305, Taiwan; maple.bt88@gmail.com (C.-F.W.); wu.chingyang@gmail.com (C.-Y.W.)
2 Department of Psychiatry, Taichung Veterans General Hospital, 4 Section, 1650 Taiwan Boulevard, Taichung 40705, Taiwan; peterhopo2@yahoo.com.tw
3 Faculty of Medicine, National Yang-Ming University, 2 Section, 155 Linong Street, Beitou District, Taipei 11221, Taiwan
4 Department of Veterinary Medicine, National Chung Hsing University, 250 Kuo Kuang Road, Taichung 40227, Taiwan; fcmao@nchu.edu.tw
5 Department of Veterinary Medicine, National Chiayi University, 580 Xinmin Road, Chiayi 60054, Taiwan; shu@mail.ncyu.edu.tw (Y.-C.S.); tsaibelle@mail.ncyu.edu.tw (H.-P.T.); pipi324615@gmail.com (H.-J.L.)
6 Department of Veterinary Medicine, School of Veterinary Medicine, National Taiwan University, 4 Section. 1 Roosevelt Road, Taipei 10617, Taiwan; yangweicheng@ntu.edu.tw (W.-C.Y.); cslin100@ntu.edu.tw (C.-S.L.)
7 College of Veterinary Medicine, Veterinary Teaching Hospital, National Chiayi University, 580 Xinmin Road, Chiayi, 60054, Taiwan
8 Ph.D. Program of Agriculture Science, National Chiayi University, 300 Syuefu Road, Chiayi 60004, Taiwan
* Correspondence: grchang@mail.ncyu.edu.tw; Tel.: +886-5-2732946
† These authors contributed equally to this work.

Received: 19 June 2020; Accepted: 12 August 2020; Published: date

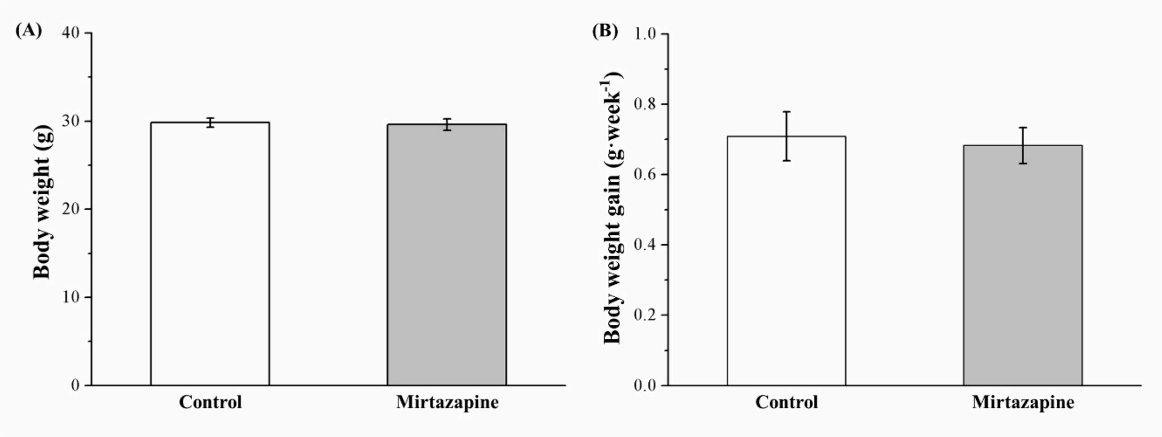

Figure 1. Changes in body weight and weekly body weight gain in the control and mirtazapine (10 mg/kg/day)-treated SD-fed mice over the 28 treatment days. We present all data in this figure as mean ± SEMs (n = 10) for both groups.

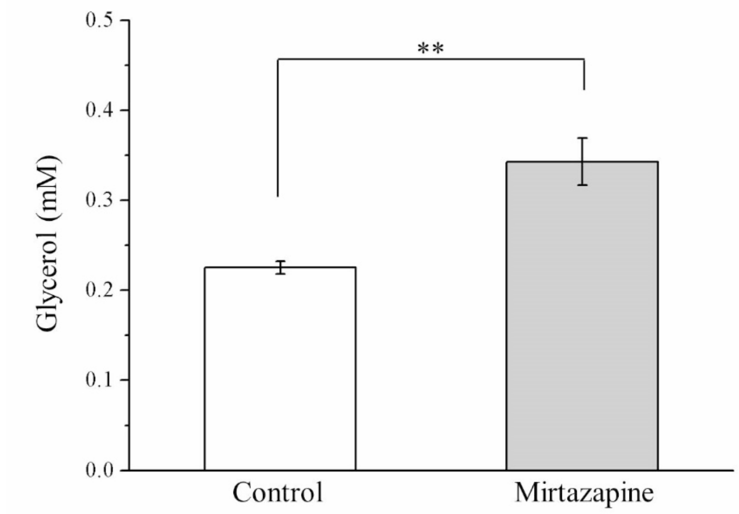

Figure 2. Changes in serum glycerol levels in the control and mirtazapine (10 mg/kg/day)-treated HFD-fed mice over the 28 treatment days. We present all data in this figure as mean ± SEMs (n = 10) for both groups. *$p < 0.05$.

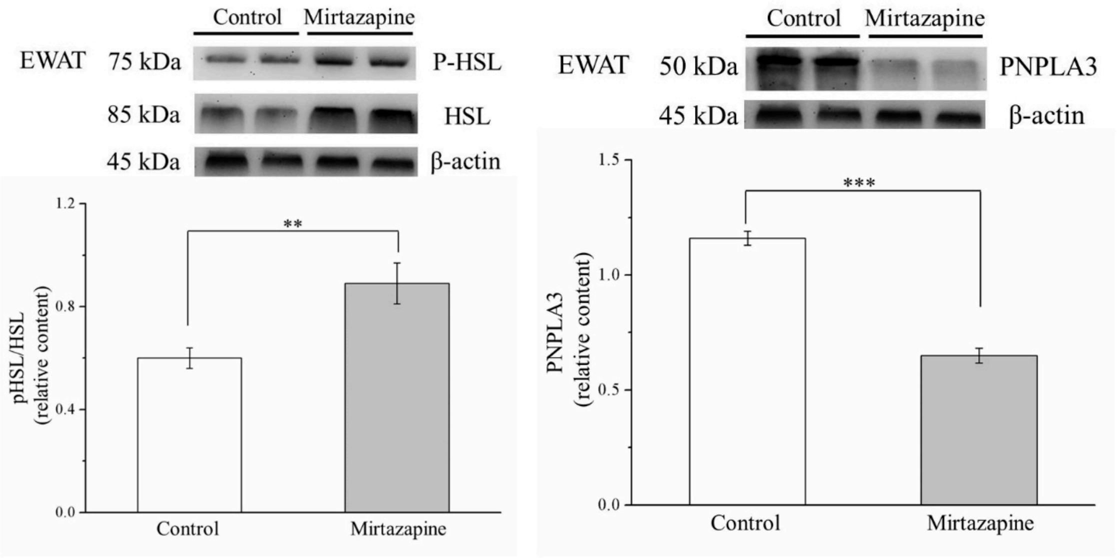

Figure 3. Changes in (**a**) HSL and (**b**) PNPLA3 in the EWAT from the control and mirtazapine (10 mg/kg/day)-treated HFD-fed mice over the 28 treatment days. We present all data in this figure as mean ± SEMs (n = 10) for both groups. *$p < 0.05$, ***$p < 0.001$.

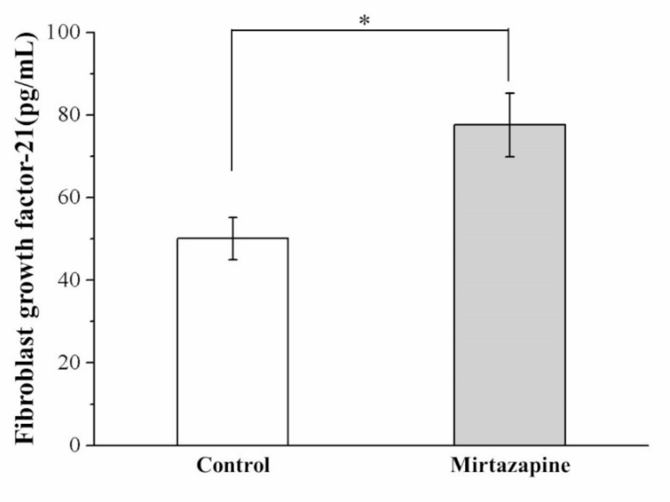

Figure 4. Changes in serum FGF-21 levels measured in the Control and Mirtazapine (10 mg/kg/day)-treated HFD-fed mice over the 28 treatment days. We present all data in this figure as mean ± SEMs (n = 10) for both groups. *$p < 0.05$.

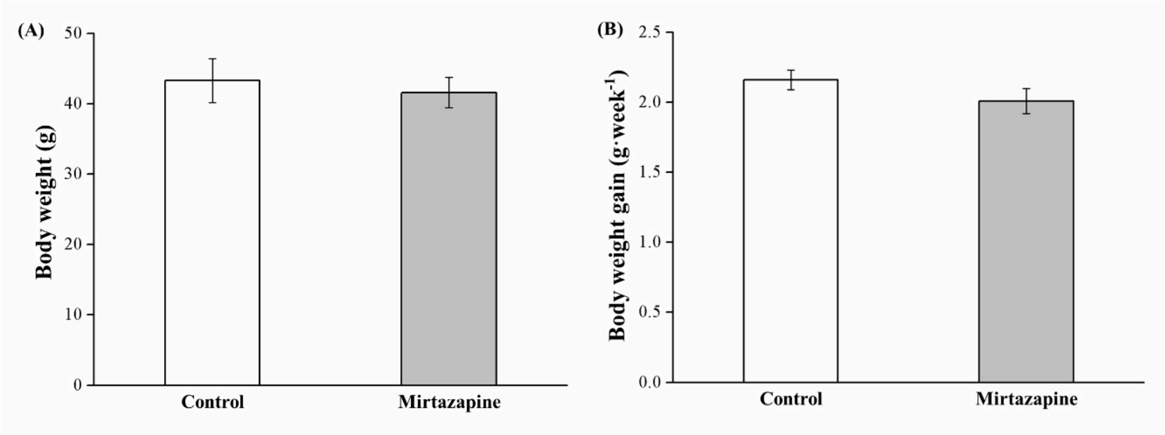

Figure 5. Changes in body weight and weekly body weight gain in the Control and Mirtazapine (2 mg/kg/day)-treated HFD-fed mice over the 28 treatment days. We present all data in this figure as mean ± SEMs (n = 10) for both groups.

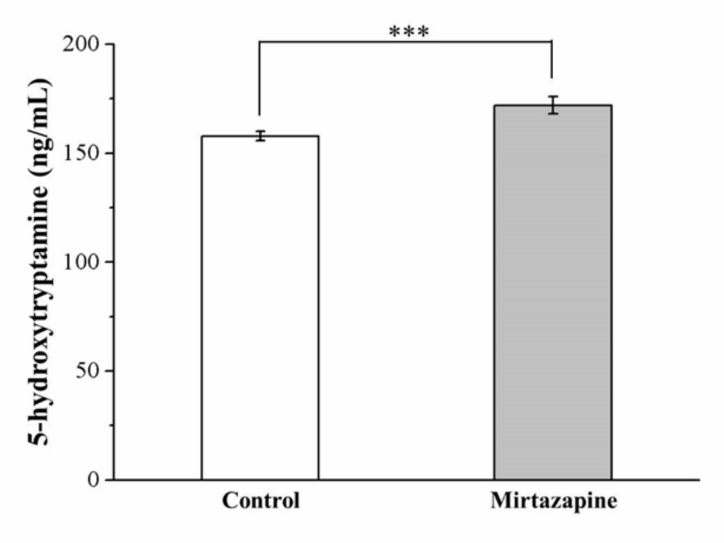

Figure 6. Changes in serum serotonin levels in the Control and Mirtazapine (10 mg/kg/day)-treated HFD-fed mice over the 28 treatment days. We present all data in this figure as mean ± SEMs (n = 10) for both groups. *p < 0.001.

© 2020 by the authors. Submitted for possible open access publication under the terms and conditions of the Creative Commons Attribution (CC BY) license (http://creativecommons.org/licenses/by/4.0/).
